# Towards an HIV cure

**DOI:** 10.7448/IAS.17.4.19479

**Published:** 2014-11-02

**Authors:** Steven Deeks

**Affiliations:** AIDS Research Institute, University of California, San Francisco, CA, USA

## Abstract

Given the challenge of delivering complex, expensive and potentially harmful antiretroviral therapy (ART) on a global level, there is intense interest in the development of short-term, well-tolerated regimens that allow individuals to interrupt therapy indefinitely without experiencing a rebound in viremia. This so-called “cure” or “remission” might be due to complete eradication of all replication-competent HIV during ART or durable host-mediated control of persistent virus in absence of ART. Recent heroic interventions such as hematopoietic stem cell transplant and very early initiation of antiretroviral therapy suggest that dramatic reductions in the reservoir size can be achieved, but that complete eradication will be difficult if not impossible to achieve. Most attempts to stimulate effective host-mediated control of HIV have failed. It is likely that for a true cure to be achieved, both approaches – reductions in the reservoir size and durable immune surveillance – will be needed, a state that is similar to that observed in “elite” controllers and post-treatment controllers. The implications for recent advances and setbacks in achieving HIV remission for future research priorities will be discussed.

